# Supplementary material for: Imaging the coupling between itinerant electrons and localised moments in the centrosymmetric skyrmion magnet GdRu2Si2
Source: Nat Commun. 2020 Nov 23;11:5925. doi: 10.1038/s41467-020-19751-4 (PMC7684290; doi:10.1038/s41467-020-19751-4)
Supplement: Supplementary file 1 — Supplementary Information [file 41467_2020_19751_MOESM1_ESM.pdf]

Supplementary Information for

Imaging the coupling between itinerant electrons and localised moments in the centrosymmetric skyrmion magnet $GdRu_2Si_2$

Yuuki Yasui,[1] Christopher J. Butler,[1] Nguyen Duy Khanh,[1] Satoru Hayami,[2,3]
Takuya Nomoto,[2] Tetsuo Hanaguri,[1] Yukitoshi Motome,[2] Ryotaro Arita,[1,2]
Taka-hisa Arima,[1,4] Yoshinori Tokura,[1,2,5] and Shinichiro Seki[2,6,7]

[1]*RIKEN Center for Emergent Matter Science, Wako, Saitama 351-0198, Japan*
[2]*Department of Applied Physics, The University of Tokyo, Bunkyo, Tokyo 113-8656, Japan*
[3]*Department of Physics, Hokkaido University, Sapporo, Hokkaido 060-0810, Japan*
[4]*Department of Advanced Materials Science, The University of Tokyo, Kashiwa, Chiba 277-8561, Japan*
[5]*Tokyo College, The University of Tokyo, Bunkyo-ku, Tokyo 113-8656, Japan*
[6]*PRESTO, Japan Science and Technology Agency (JST), Kawaguchi 332-0012, Japan*
[7]*Institute of Engineering Innovation, The University of Tokyo, Bunkyo, Tokyo 113-8656, Japan*

(Dated: October 15, 2020)

Supplementary Note 1. Spectroscopic-imaging STM on Gd-terminated surface

We conducted spectroscopic-imaing scanning tunnelling microscopy (SI-STM) experiments on the Gd-terminated surface and found that the dI/dV spectrum and the spatial distribution of the electronic state exhibit more complex behaviours than those of the Si-terminated surface. Spatially averaged dI/dV spectra are shown in Supplementary Figs. 1(a) and (b). To clarify how the spectrum changes with magnetic field, the value of dI/dV at -6 mV as a function of applied magnetic field is plotted in Supplementary Fig. 1(c). The dI/dV value develops rapidly as increasing the magnetic field and takes a kink at 1.4 T followed by a plateau. The plateau extends up to 2.2 T, which is the transition field between Phase I and II in the bulk. Then, dI/dV at -6 mV shows a dip at 2.3 T, which corresponds to Phase II. In Phase III, right above the skyrmion phase, dI/dV at -6 mV takes slightly higher value than that of the plateau and continuously decreases with increasing the field. A step decrease is observed at the boundary between Phase III and the FP phase.

The bulk magnetic phase boundaries manifest themselves as anomalies in the field dependence of the dI/dV spectrum of the Gd-terminated surface as well, even though the appearance is different from that of the Si-terminated surface. The major difference between the two terminations appears in Phase I. The spectrum varies only slightly below 2.2 T for the Si-terminated surface. In contrast, a clear kink at 1.4 T

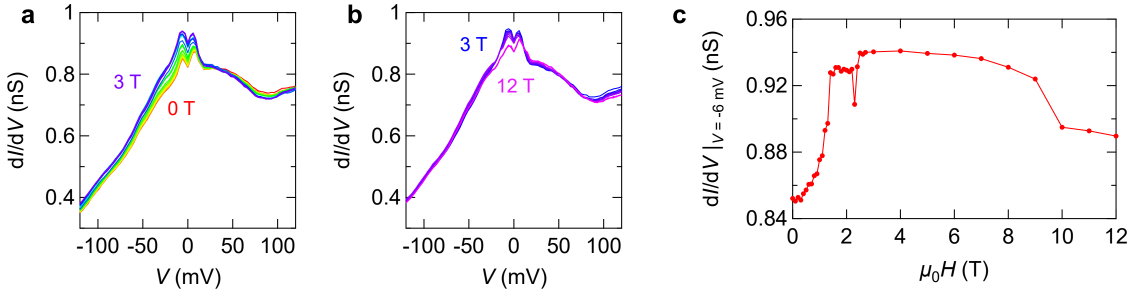

Supplementary Figure 1. Spatially averaged dI/dV curves of the Gd-terminated surface **a** below 3 T and **b** above 3 T measured at 1.5 K. V_s = 120 mV, I_s = 100 pA, and V_{mod} = 3 mV. **c**, Magnetic field dependence of the value of dI/dV at $V = -6$ mV.

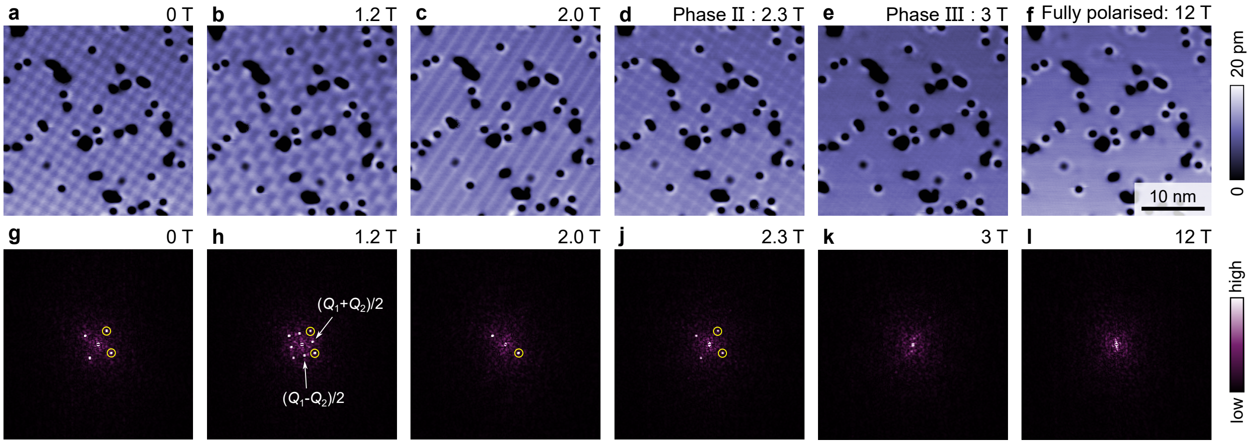

Supplementary Figure 2. **a-f**, Constant-current topographic images of the Gd-terminated surface measured at 1.5 K. V_s = 120 mV and I_s = 100 pA. **g-l**, Fourier transforms of the filtered topographic images z_{filtered}. Peaks marked with yellow circles correspond to $\mathbf{Q}_1$ or $\mathbf{Q}_2$ modulations.

in Supplementary Fig. 1(c) suggests that the Gd-terminated surface possesses multiple magnetic states in the bulk Phase I. This feature may originate from surface effects, because the magnetic Gd layer is directly exposed to the vacuum for the Gd termination, whereas it is sandwiched by RuSi layers for the Si-terminated surface.

We further investigated Phase I of the Gd-terminated surface by means of constant-current topographic imaging and found three different periodic structures, as shown in Supplementary Figs. 2(a)-(c). The constant-current topographic images represent the integrated local density of states (LDOS) from the Fermi energy to the setup sample bias (0 to 120 meV for these measurements) on top of surface corrugations. Since atomic corrugations are not detected, the topographs here should be governed by the spatial structures of the LDOS and indicate that the Gd-terminated surface hosts three distinct states within bulk Phase I. Topographic images for the other phases are also shown in Supplementary Figs. 2(d)-(f) for comparison.

We found that deep defects in Supplementary Fig. 2(a)-(f) dominate the Fourier-transformed images and hide signals associated with the periodic structures. Therefore, the topographic images are filtered using the following relation before Fourier analysis.

$$z_{\text{filtered}}(\mathbf{r}) \equiv \begin{cases} z(\mathbf{r}) & \text{for } |z(\mathbf{r}) - z_{\text{med}}| \leq 0.3 z_{\text{stdev}} \\ z_{\text{med}} & \text{for } |z(\mathbf{r}) - z_{\text{med}}| > 0.3 z_{\text{stdev}}, \end{cases} \tag{S1}$$

where z_{filtered} is the filtered height, z is the measured height in the topographic images, z_{med} is the median value of each topograph, z_{stdev} is the standard deviation of each topograph, and $\mathbf{r}$ is the lateral position. Fourier transforms (FTs) of the filtered topographs are shown in Supplementary Figs. 2(g)-(l). Modulation vectors are observed at $\mathbf{Q}_1$ and $\mathbf{Q}_2$ (yellow circles), and $\frac{1}{2}(\mathbf{Q}_1 \pm \mathbf{Q}_2)$ (white arrows). The $\mathbf{Q}_1$- and $\mathbf{Q}_2$-related modulation vectors suggest that bulk magnetic structure plays an important role. We speculate that additional surface effects may modify the magnetic structure on the Gd-terminated surface, resulting in the multiple states within bulk Phase I. Details are yet to be investigated.

Supplementary Note 2. Additional data supporting the main observation

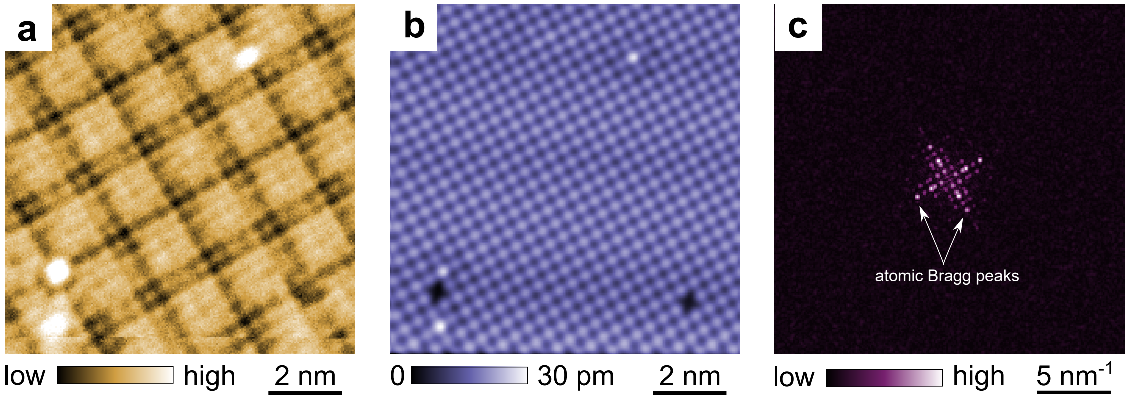

Supplementary Figure 3. **a**, $L(\boldsymbol{r}, E = -20\ \mathrm{meV}) = \frac{dI}{dV} / \frac{I}{V}$ map with higher spatial resolution measured at $\mu_0 H = 2.3$ T and $T = 1.5$ K (Phase II, Skyrmion). No additional fine features are observed. The scan was done from bottom to top. The discontinuity at the bottom of the image shows that the skyrmion structure slides. Such motion is often observed soon after magnetic field was swept. **b**, Simultaneously measured topograph. Slides in atomic lattice was not observed. This suggests that the magnetic structures are not pinned to surface impurities. The setup sample bias voltage was $V_s = 100$ mV, tunnelling current was $I_s = 100$ pA, and the bias modulation amplitude was $V_{\mathrm{mod}} = 5$ mV. **c**, Fourier transform of (a).

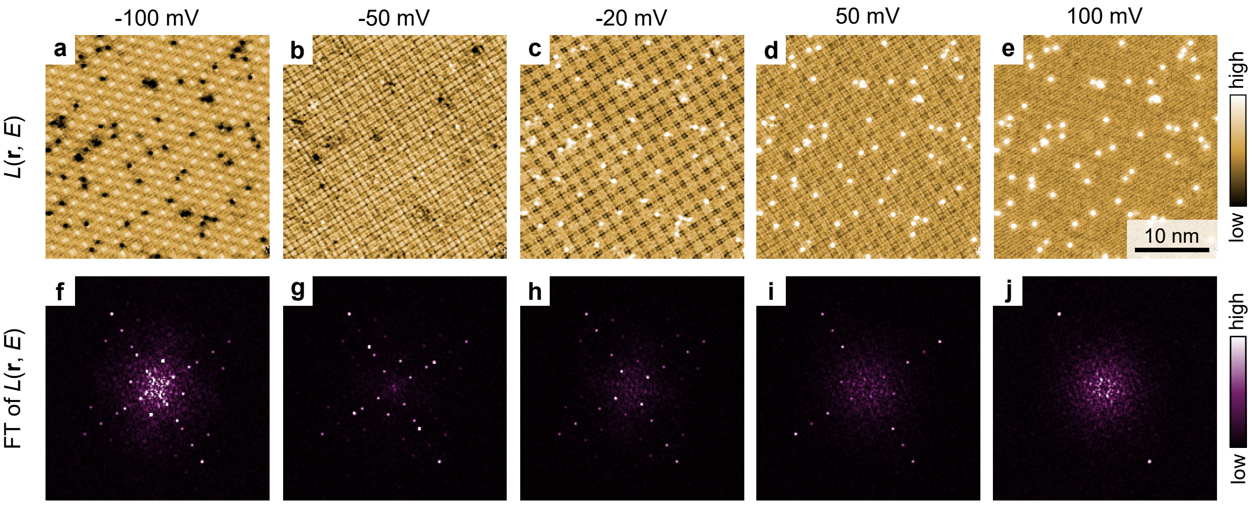

Supplementary Figure 4. $L(\boldsymbol{r}, E) = \frac{dI}{dV} / \frac{I}{V}$ maps at different slices of bias voltages measured at $\mu_0 H = 2.3$ T and $T = 1.5$ K (Phase II, Skyrmion). The position of thee Fourier spots do not depend on bias voltages while their intensity changes. $V_s = 100$ mV, $I_s = 100$ pA, and $V_{\mathrm{mod}} = 5$ mV.

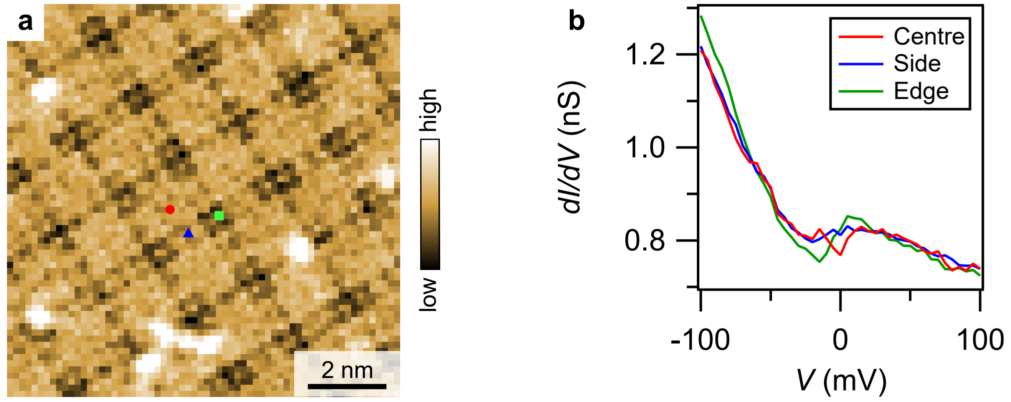

Supplementary Figure 5. **a**, $L(\boldsymbol{r}, E = -20$ meV) map measured at $\mu_0 H = 2.3$ T and $T = 1.5$ K (Phase II, Skyrmion). Characteristic positions are marked with red circle (centre), blue triangle (side), and green square (edge). **b**, dI/dV spectra at different potions. $V_s = 100$ mV, $I_s = 100$ pA, and $V_{mod} = 5$ mV.

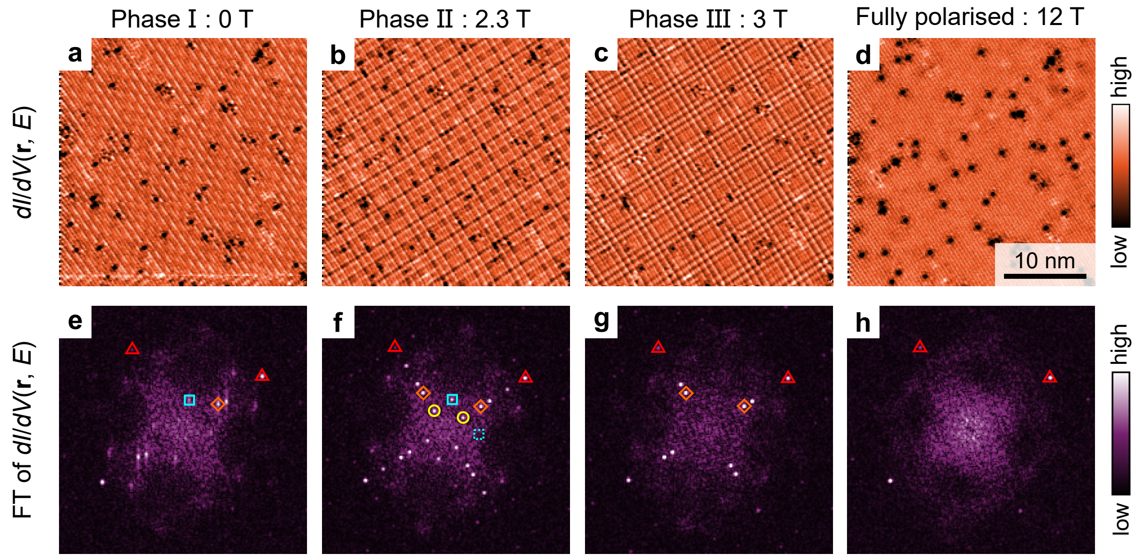

Supplementary Figure 6. dI/dV maps at $E = -20$ meV measured at $T = 1.5$ K. These are the same sets of data as Fig. 3 in the main text but without normalisation. The normalisation does not affect to the Fourier Q-components. $V_s = 100$ mV, $I_s = 100$ pA, and $V_{mod} = 5$ mV.

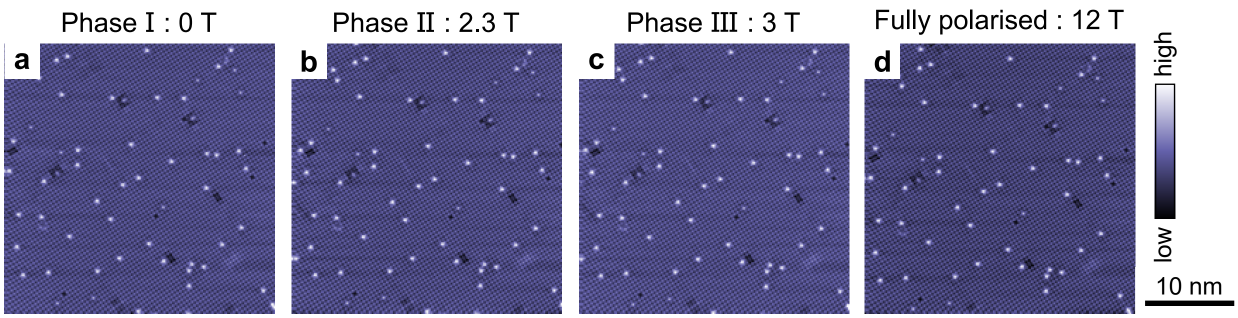

Supplementary Figure 7. Topographic images simultaneously measured with Fig. 3a-d of the main text. The impurities as well as the scanning tip did not change during the measurement. V_s = 100 mV and I_s = 100 pA.

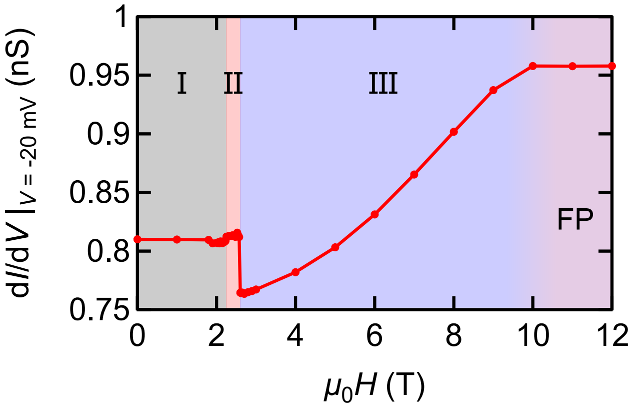

Supplementary Figure 8. Magnetic field dependence of dI/dV at −20 mV. First-order like transitions across Phase II and continuous change in Phase III are also seen at $V = -20$ mV. This behaviour does not depend on bias voltage as presented in Fig. 3k in the main text.

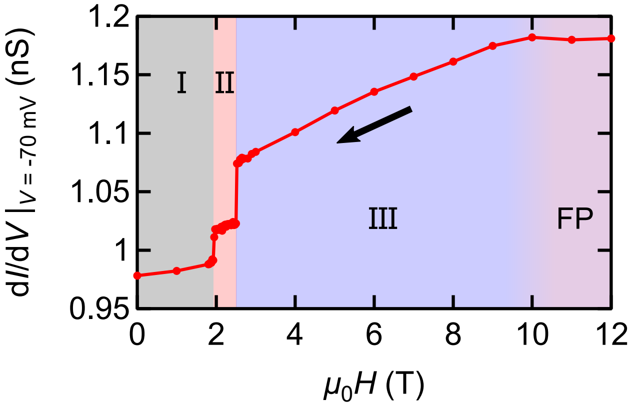

Supplementary Figure 9. Magnetic field dependence of dI/dV at −70 mV for magnetic field swept down from 12 T. The transition out from Phase II is slightly shifted to lower value due to its first-order transition.

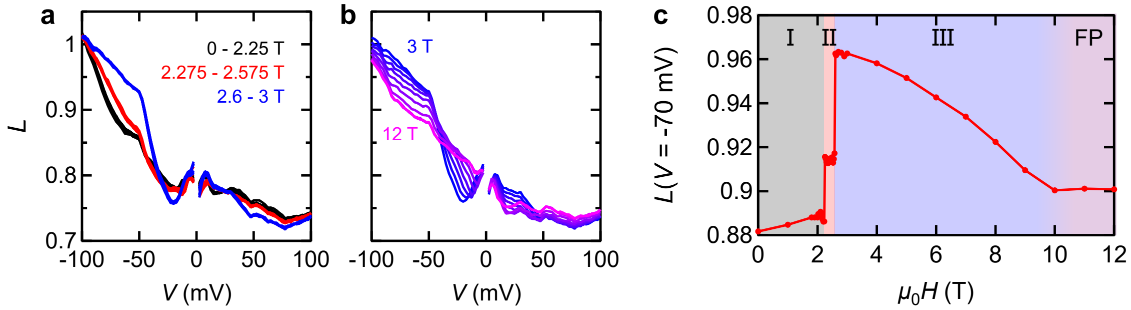

Supplementary Figure 10. **i** and **j**, Spatially averaged normalised conductance $L = \frac{dI}{dV}/\frac{I}{V}$ curves **i** below 3 T and **j** above 3 T. $V_s = 100$ mV, $I_s = 100$ pA, and $V_{mod} = 2.5$ mV. **k**, Magnetic field dependence of the value of L at $V = -70$ mV.

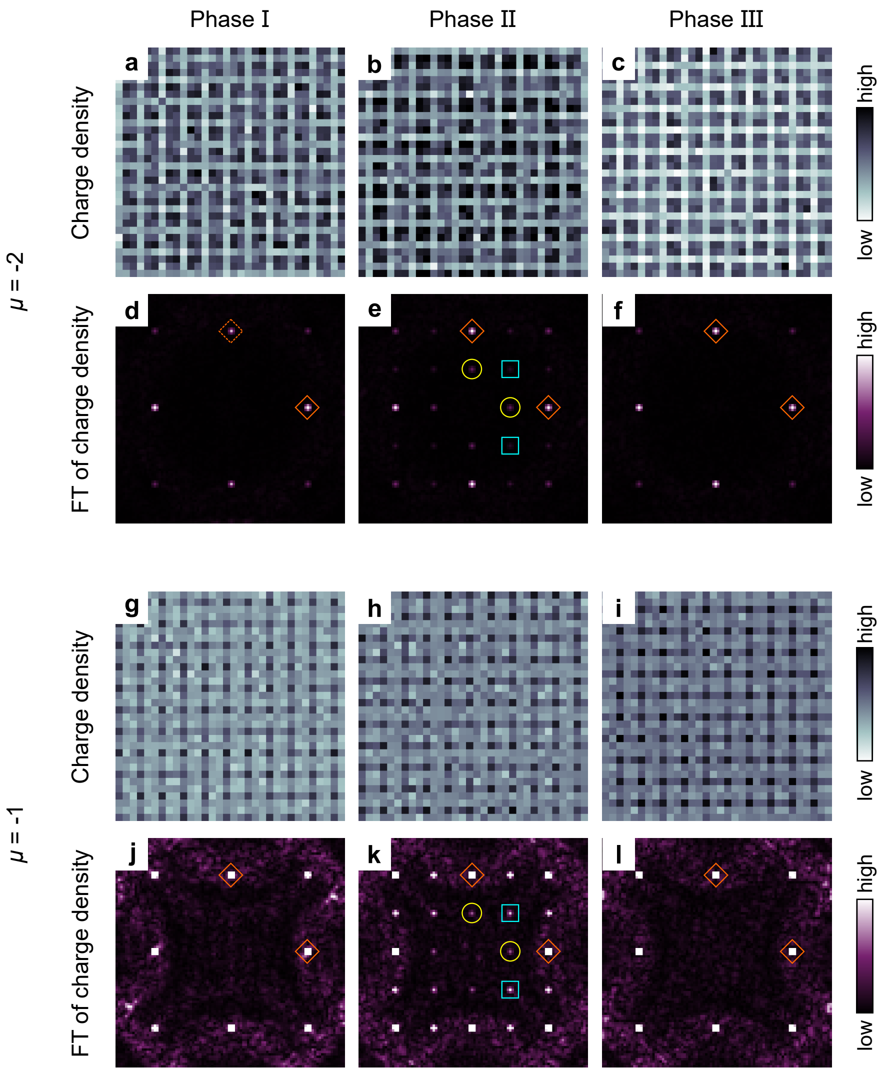

Supplementary Figure 11. Charge density modulations calculated for different chemical potentials μ. $\mu = -3$ is used for Fig. 4 in the main text. The Fourier Q-components do not depend on parameters we choose.
